# Kinase Inhibitors that Increase the Sensitivity of Methicillin Resistant *Staphylococcus aureus* to β-Lactam Antibiotics

**DOI:** 10.3390/pathogens4040708

**Published:** 2015-10-22

**Authors:** Jay Vornhagen, Kellie Burnside, Christopher Whidbey, Jessica Berry, Xuan Qin, Lakshmi Rajagopal

**Affiliations:** 1 Department of Pediatric Infectious Diseases, University of Washington School of Medicine, Seattle, WA 98195, USA; E-Mails: jay.vornhagen@seattlechildrens.org (J.V.); Kellie.Howard@labcorp.com (K.B.); christopher.whidbey@pnnl.gov (C.W.); 2 Seattle Children’s Research Institute, 1900 Ninth Avenue, Seattle, WA 98101, USA; E-Mails: jessica.berry@seattlechildrens.org (J.B.); xuan.qin@seattlechildrens.org (X.Q.); 3 Department of Global Health, University of Washington School of Public Health, Seattle, WA 98195, USA

**Keywords:** serine/threonine kinase, sulfonamides, antibiotics, inhibition, mouse

## Abstract

*Staphylococcus aureus* are Gram-positive bacteria that are the leading cause of recurrent infections in humans that include pneumonia, bacteremia, osteomyelitis, arthritis, endocarditis, and toxic shock syndrome. The emergence of methicillin resistant *S. aureus* strains (MRSA) has imposed a significant concern in sustained measures of treatment against these infections. Recently, MRSA strains deficient in expression of a serine/threonine kinase (Stk1 or PknB) were described to exhibit increased sensitivity to β-lactam antibiotics. In this study, we screened a library consisting of 280 drug-like, low-molecular-weight compounds with the ability to inhibit protein kinases for those that increased the sensitivity of wild-type MRSA to β-lactams and then evaluated their toxicity in mice. We report the identification of four kinase inhibitors, the sulfonamides ST085384, ST085404, ST085405, and ST085399 that increased sensitivity of WT MRSA to sub-lethal concentrations of β-lactams. Furthermore, these inhibitors lacked alerting structures commonly associated with toxic effects, and toxicity was not observed with ST085384 or ST085405 *in vivo* in a murine model. These results suggest that kinase inhibitors may be useful in therapeutic strategies against MRSA infections.

## 1. Introduction

Bacterial infections remain a significant cause of morbidity and mortality in humans*. Staphylococcus aureus* are Gram-positive bacteria that cause clinically significant, and sometimes reoccurring, infections [1]. Although *S. aureus* are frequently found as colonizers in the nose and skin in about 20% of the human population, severe infections due to *S. aureus* include pneumonia, bacteremia, osteomyelitis, arthritis, endocarditis, and toxic shock syndrome. Furthermore, patients diagnosed with hyper IgE (Jobs) syndrome or chronic granulomatous diseases (CGD) are predisposed to frequent and life-threatening *S. aureus* infections. While antibiotic therapy is currently used to treat *S. aureus* infections, the emergence of antibiotic resistant strains, such as those resistant to methicillin and vancomycin, are rapidly exhausting available treatment options [2,3]. MRSA infections are commonly treated with non-β-lactam antibiotics, such as clindamycin and co-trimoxaole [4]. Intravenous administration, toxicity, and limited penetration of glycopeptide antibiotics into deeper tissues impose additional constraints on treatment [5].

MRSA strains lacking serine/threonine kinase (∆*stk1*) were described to exhibit increased sensitivity to β-lactams [6,7]. Furthermore, the kinase activity of Stk1 was shown to be important for antibiotic resistance as complementation of ∆*stk1* with only the kinase domain restored WT level antibiotic resistance [7]. Therefore, we screened a kinase inhibitor library to identify compounds that could increase the sensitivity of MRSA to β-lactam antibiotics. We report the identification of four sulfonamide kinase inhibitors, ST085384, ST085404, ST085405, and ST085399, that increased sensitivity of WT MRSA to sub-lethal concentrations of β-lactams. These findings suggest that kinase inhibitors may be promising in therapeutic strategies against MRSA infections.

## 2. Results

### 2.1. Kinase Inhibitors Increase Sensitivity of MRSA to β-Lactam Antibiotics, Such as Nafcillin

We hypothesized that inhibition of Stk1 by kinase inhibitors should increase the sensitivity or minimal inhibitory concentration (MIC) of MRSA to β-lactam antibiotics. To this end, using methods described [8], we first derived Δ*stk1* mutants from CA-MRSA strains linked to sequential and overlapping epidemics in the United States (USA300 LAC and USA400 MW2 [9]). Antibiotic susceptibility testing was performed using standards established by the Clinical and Laboratory Standards Institute (CLSI, [10]). Consistent with previous findings [6,7], we observed that ∆*stk1* mutants derived from MRSA strains MW2 and LAC showed a dramatic increase in susceptibility to β-lactams (lower MIC, see Table 1). The above results further confirmed that MRSA ∆*stk1* mutants are more sensitive to β-lactam antibiotics and provided support for the identification of kinase inhibitors.

**Table 1:** Role of Stk1 in β-lactam resistance of methicillin-resistant *S. aureus* (MW2 and LAC).

MIC (µg/mL)	PEN	AMP	NAF	CXM	CAZ	FEP	IPM
Newman (MSSA)	0.094	0.25	0.25	1.5	16	4	0.032
MW2	48	24	32	>256	>256	>256	1
MW2∆*stk1*	12	12	2	6	32	8	0.125
LAC	48	32	16	>256	>256	>256	0.75
LAC∆*stk1*	24	24	4	6	12	4	0.06

PEN = Penicillin; AMP = Ampicillin; NAF = Nafcillin; CXM = Cefuroxime; CAZ = Caftazidime; FEP = Cefepime; IMI = Imipenem. Minimal inhibitory concentration (MIC) was determined using either Etest strips (AB Biodisk) or growth in liquid broth. For MIC determination in liquid growth, overnight cultures of MRSA strains were inoculated 1:20 in Typtic Soy Broth (TSB) containing different antibiotic concentrations.

**Figure 1:**
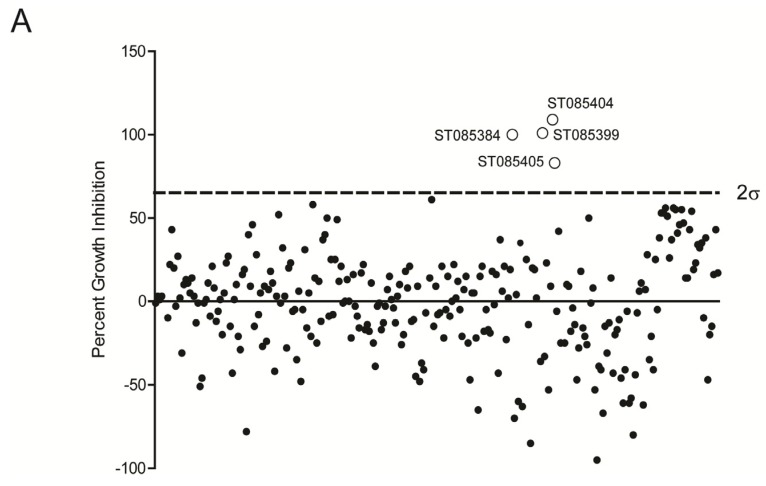

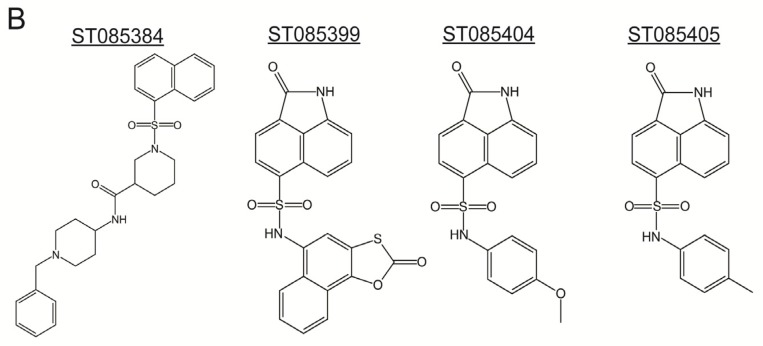
Identification of sulfonamides that increase the sensitivity of MRSA to Nafcillin (NAF). (**A**) Cultures of MRSA WT LAC were grown in the presence of sub-lethal concentration of NAF with various sulfonamides to identify putative kinase inhibitors. Four compounds (ST085384, ST085399, ST085404, and ST085405) caused growth inhibition greater than two standard deviations of the data set; and (**B**) structures of the four identified sulfonamides.

We next screened a library consisting of 280 drug-like, low molecular weight compounds that can inhibit protein kinases (http://www.timtec.net/kinase-modulators-actitarg-k-library.html) for their ability to increase the sensitivity of wild type (WT) MRSA to β-lactams antibiotics. Briefly, WT MRSA (LAC) was grown overnight in the presence of sub-lethal concentrations of NAF (4 µg/mL) in the presence of 40 µg/mL of the respective kinase inhibitor. Controls included MRSA (LAC) grown in the presence of the kinase inhibitor alone, NAF alone, or media alone. As controls, LACΔ*stk1* was also grown in the presence of the antibiotic, the kinase inhibitor, or media only. The data are shown as % inhibition compared to growth of WT MRSA LAC in the absence of the inhibitor (Figure 1A). These studies revealed that four inhibitors namely ST085384 ([N-(1benzylpiperidin-4-yl)-1-(naphthalen-1ylsulfonyl)piperidine-3carboxamide], ST085399 (N-(4-methylphenyl)-2-oxo-1H-benzo[cd]indole-6-sulfonamide), ST085404 (2-oxo-N-(2-oxonaphtho[2,1-d][1,3]oxathiol-5-yl)-1,2-dihydrobenzo[cd]indole-6-sulfonamide), ST085405 (N-(4-methoxyphenyl)-2-oxo-2H-naphtho[1,8-bc]thiophene-6-sulfonamide), increased sensitivity of WT MRSA to β-lactams (>66% inhibition Figure 1A, see Figure 1B for structures). These kinase inhibitors also did not inhibit growth of the MRSAΔ*stk1* mutant (*i.e.*, ≤0% growth inhibition).

**Figure 2:**
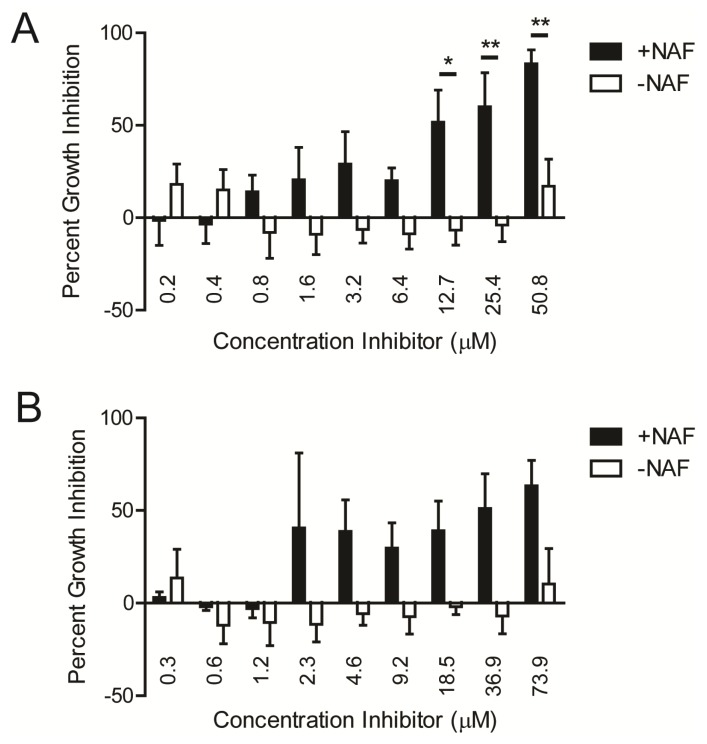

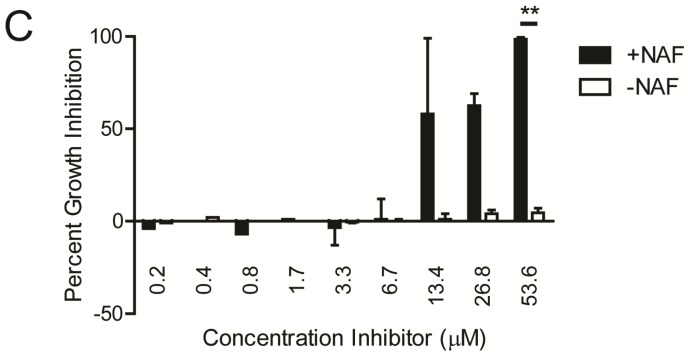
Kinase inhibitors induce a dose-dependent increase in the sensitivity of MRSA to NAF *in vitro*. Cultures of MRSA WT LAC were grown in the presence or absence of NAF (4 µg/mL) with various concentrations of (**A**) ST085384; (**B**) ST085399; or (**C**) staurosporine. The concentration required for 50% growth inhibition for ST085384 (12.7 µM) was comparable to that of staurosporine (13.4 µM). At the highest concentration used, the kinase inhibitors were at ~25 µg/mL. * *p* < 0.05; ** *p* < 0.01; Bonferroni Multiple Comparisons following Two-way ANOVA; *n* = 2–3).

Using ST085384 (Figure 2A) and ST085399 (Figure 2B), we confirmed that growth inhibition of MRSA LAC was observed only in the presence of the β-lactam (Figure 2A,B). At 12.7 µM, ST085384 inhibited growth of MRSA by 52% (Figure 2A), which is comparable to the 58% inhibition observed with staurosporine (a general and potent kinase inhibitor) at a similar concentration (Figure 2C, 13.4 µM). Similar results were observed with MRSA MW2.

**Figure 3:**
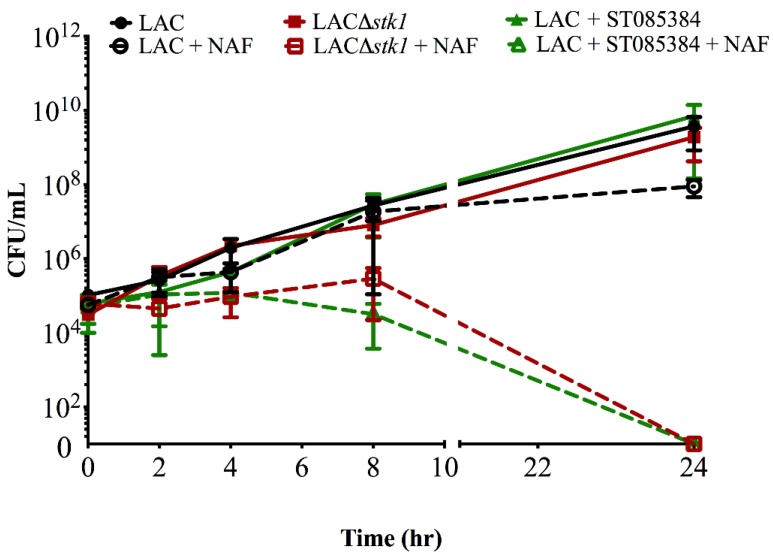
The kinase inhibitor ST085384 increases sensitivity of MRSA LAC to the bactericidal activity of Nafcillin (NAF).

To confirm that the decrease in optical density observed on treatment of MRSA with kinase inhibitor and NAF correlated with decreased bacterial CFU, we performed a time-to-kill analysis, as described [11,12]. Briefly, 10^4^ CFU of WT MRSA (LAC) was grown for 24 h in either media alone, antibiotic NAF alone (4 µg/mL), kinase inhibitor alone (40 µg/mL), or kinase inhibitor containing NAF. For enumeration of viable bacterial CFU, aliquots were serially diluted and plated at 0, 2, 4, 8, and 24 h post-inoculation. As a control, approximately 10^4^ CFU/mL of LACΔ*stk1* (which is sensitive to NAF) was added to either media alone or media containing NAF (4 µg/mL). The results shown in Figure 3A confirm that treatment of MRSA LAC with the kinase inhibitor and antibiotic NAF was bactericidal, whereas treatment with either only the kinase inhibitor or only the antibiotic NAF did not confer bactericidal activity. Additionally, the bactericidal activity of NAF to MRSA LAC treated with ST085384 is similar to that observed with the β-lactam sensitive MRSA LACΔ*stk1* treated with NAF.

The MRSA strain LAC was grown overnight from single colony. The next morning, approximately 10^4^ CFU/mL of LAC was added to 500 µL of either TSB (shown below as LAC), TSB containing NAF (4 µg/mL, shown below as LAC + NAF), TSB containing kinase inhibitor ST085384 (40 µg/mL, shown below as LAC + ST085384), or TSB containing both NAF (4 µg/mL) and ST085384 (40 µg/mL); shown below as LAC + ST085384 + NAF, see green dashed line. As a control, approximately 10^4^ CFU/mL of LACΔ*stk1* (which is sensitive to NAF) was added to either 500 µL of TSB (see LACΔ*stk1*) or 500 µL of TSB containing NAF (4 µg/mL, see LACΔ*stk1* + NAF, red dashed line). All cultures were grown in a 37 °C shaker at 220 rpm. Aliquots of the media were removed immediately after subculture (T_0_) and at 2, 4, 8, and 24 h post-inoculation and were serially diluted and plated on TSA. Plates were incubated for 24–48 h at 37 °C and viable CFU were counted. The experiment was repeated three times. Note that NAF is bactericidal to MRSA LAC only in the presence of the kinase inhibitor ST085384 and is similar to that observed with MRSA LACΔ*stk1* treated with NAF (see red and green dashed lines).

**Figure 4:**
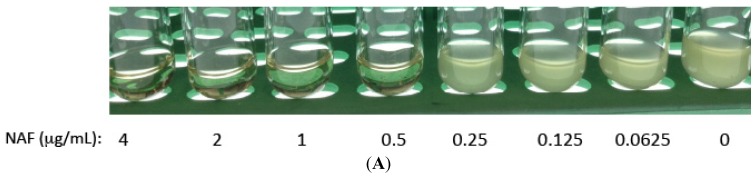

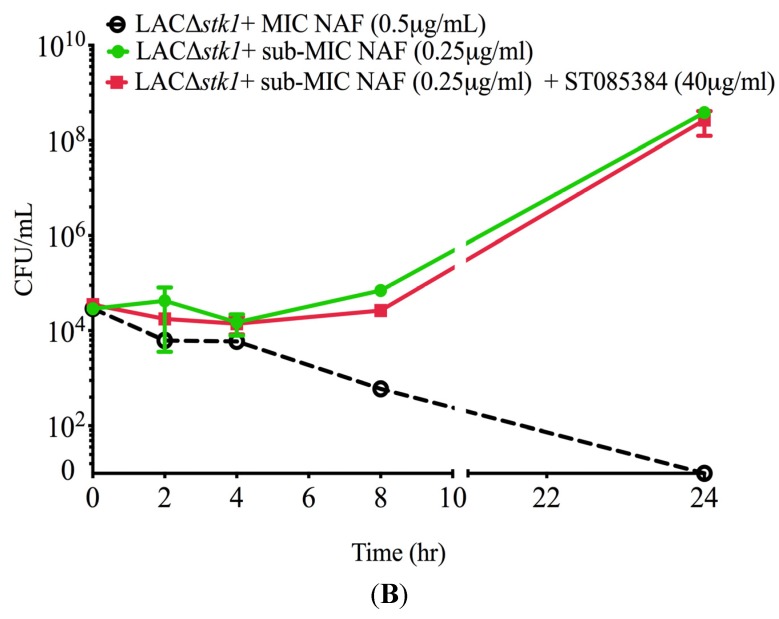
The kinase inhibitor ST085384 does not increase sensitivity of MRSA LAC∆*stk1* to the bactericidal activity of Nafcillin (NAF). (**A**) The MRSA strain LAC∆*stk1* was grown overnight from a single colony. The next morning, approximately 10^4^ CFU/mL of LAC∆*stk1* was added to 1 mL TSB without antibiotics or 500 µL of TSB containing two-fold serial dilutions of Nafcillin from 0.0625 µg/mL to 4 µg/mL and was incubated O/N at 37 °C with shaking. The MIC of NAF for LAC∆*stk1* under these conditions was determined to be 0.5 µg/mL (see loss of turbidity or lack of growth in Panel A); (**B**) approximately 10^4^ CFU/mL of LAC∆*stk1* was added to 500 µL of either TSB or TSB containing MIC NAF (0.5 µg/mL, indicated as LAC∆*stk1* + MIC NAF), TSB containing sub-MIC NAF (0.25 µg/mL, indicated as LAC∆*stk1* + sub-MIC NAF), and TSB containing sub-MIC NAF (0.25 µg/mL + kinase inhibitor ST085384 (40 µg/mL), indicated as LAC∆*stk1* + sub-MIC NAF + ST085384). All cultures were grown in a 37 °C shaker at 220 rpm. Aliquots of the media were removed immediately after subculture (T_0_) and at 2, 4, 8, and 24 h post-inoculation, and were serially diluted and plated on TSA. Plates were incubated for 24–48 h at 37 °C and viable CFU were counted. The experiment was repeated twice. Note that ST085384 did not increase sensitivity of LACΔ*stk1* to NAF.

To determine if ST085384 had potential off-target effects that also increased sensitivity of MRSA LACΔ*stk1* to NAF, we compared growth of LACΔ*stk1* in the presence of sub-MIC NAF with and without the kinase inhibitor ST085384. To this end, we first determined the MIC and sub-MIC of NAF for LACΔ*stk1* under the time to kill assay conditions. To this end, approximately 10^4^ CFU/mL of LACΔ*stk1* was added to 500 μL of TSB containing two-fold serial dilutions of Nafcillin. Under these conditions, we determined that the MIC of NAF for LACΔ*stk1* to be 0.5 μg/mL, see Figure 4A). The MIC of NAF for LAC∆*stk1* under the time to kill assay conditions was lower than that shown in Table 1, likely due to different inoculum densities. Subsequently, ~10^4^ CFU/mL of LACΔ*stk1* was added to 500 μL of either TSB or TSB containing MIC NAF (0.5 µg/mL), TSB containing sub-MIC NAF (0.25 µg/mL) and TSB containing sub-MIC NAF (0.25 µg/mL + kinase inhibitor ST085384 (40 µg/mL)). Aliquots of the media were removed immediately after subculture (T_0_) and at 2, 4, 8, and 24 h post-inoculation and were serially diluted and plated in duplicate on TSA. Note that ST085384 did not increase the sensitivity of LACΔ*stk1* to NAF. Collectively, these data confirm that the kinase inhibitor used in this study increases sensitivity of WT MRSA to ß-lactam antibiotics.

### 2.2. Kinase Inhibitors Decrease Stk1 Autophosphorylation

**Figure 5:**
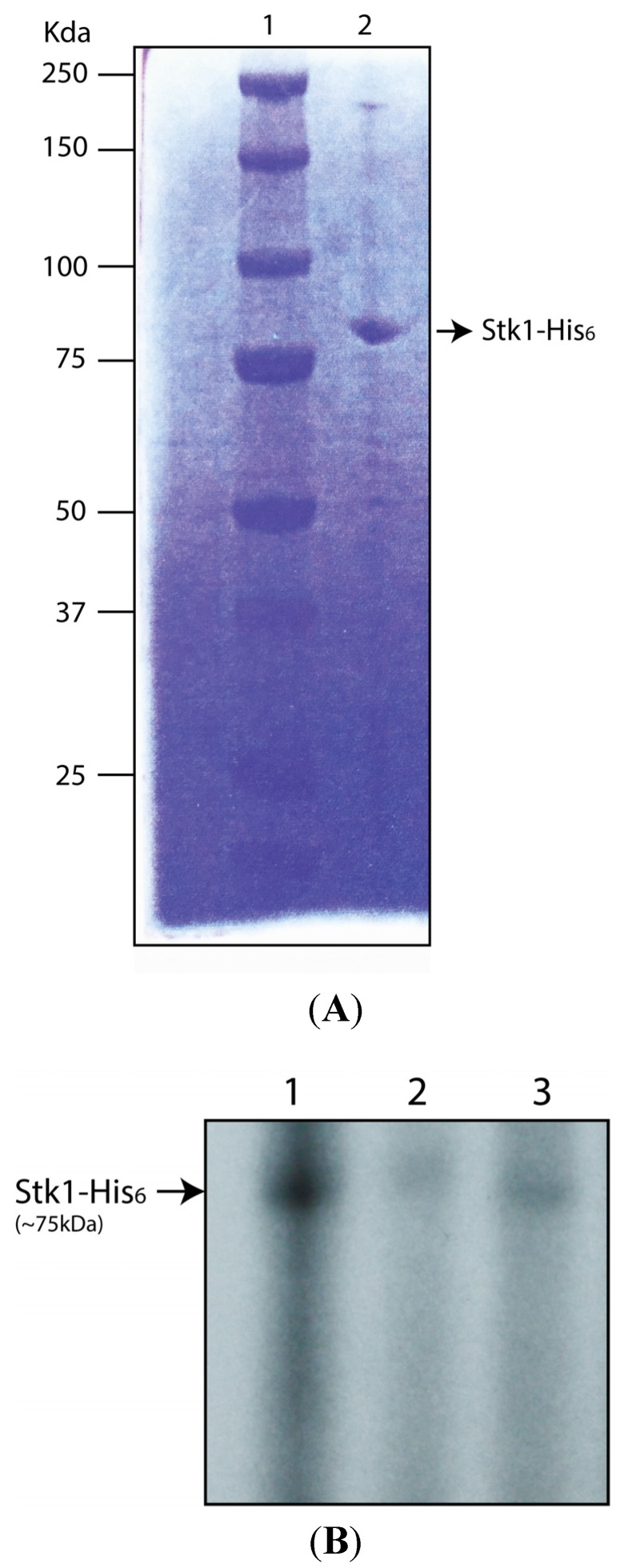
Kinase inhibitor ST085384 decreases Stk1 autophosphorylation *in vitro*. (**A**) S. *aureus* Stk1-His_6_ protein was purified as described in the methods, and an aliquot of the purified protein was resolved on 10% SDS-PAGE and stained with Coomassie Brilliant Blue. Lane 1 represents pre-stained protein standard (BioRad) and Lane 2 presents Stk1-His_6_ (~1 µg); and (**B**) autophosphorylation assays of Stk1 was performed using *S. aureus* Stk1-His_6_(~1 µg) in kinase buffer containing 10 µCi [γ ^32^P]-ATP and either DMSO control (lane 1) or Staurosporine (2 µM, lane 2) or ST085384 (2 µM, lane 3). Samples were resolved on 12% SDS-PAGE followed by autoradiography. Note that autophosphorylation of Stk1 is decreased in the presence of staurosporine (lane 2) and ST085384 (lane 3).

To confirm that kinase inhibitors such as ST085384 can inhibit Stk1 from *S. aureus*, we performed autophosphorylation assays. To this end, we cloned the coding sequence of the *stk1* gene from *S. aureus* into the expression vector pET32ck [13] to generate a C-terminal His-tagged fusion. Although we used genomic DNA from *S. aureus* RN4420 as the template for *stk1* cloning, we confirmed that the coding sequence of the *stk1* gene in *S. aureus* RN4220 [14] is 100% identical to that found in MRSA USA300 [15]. Stk1 protein was then purified and an aliquot was analyzed on 10% SDS-PAGE followed by Coommasie staining (Figure 5A). Then autophosphorylation asssays were performed in buffer containing 10 µCi [γ ^32^P]-ATP and either DMSO (control) or Staurosporine (2 µM) or ST085384 (2 µM) for 15 min. Subsequently, all samples were separated by SDS-PAGE followed by autoradiography. Figure 5B shows that autophosphorylation of Stk1 is decreased in the presence of Staurosporine or ST085384 (compare lanes 2 and 3 to 1). These data indicate that the kinase inhibitor ST085384 and Staurosporine can inhibit the activity of *S. aureus* Stk1.

### 2.3. The Kinase Inhibitor ST085384 Is Tolerated in Mice

**Figure 6:**
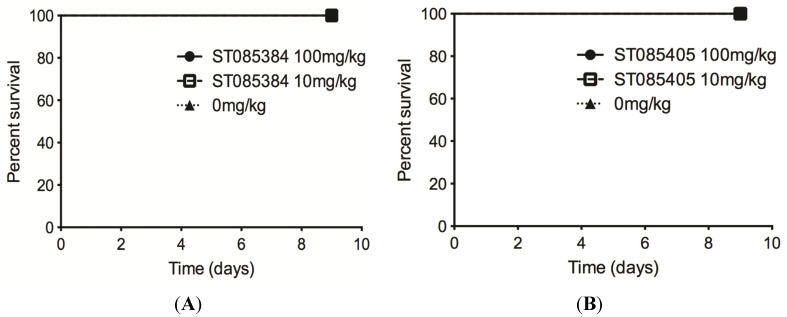
The kinase inhibitors ST085384 and ST085405 are tolerated in mice. Survival of mice injected with 100 mg/kg (black circle and solid line), 10 mg/kg (hollow square and dashed line), or vehicle only (0 mg/kg; black triangle and dotted line) of (**A**) ST085384 or (**B**) ST085405 was monitored for nine days post-injection. Note that all mice survived.

Bioinformatic analysis using the Mobyle FAF-drugs2 software [16] indicated that “alerting structures” commonly associated with toxic effects were not present in ST085384, ST085404, ST085405, or ST085399. Additionally, ST085384, ST085404, ST085405, ST085399 did not exhibit any violations to Lipinski’s rule of five that are important for solubility and permeability of compounds in drug discovery and development [17]. Therefore, we were next interested to determine if the kinase inhibitor/s is tolerated by mice. We chose to test this with the kinase inhibitors ST085384 and ST085405. To this end, we administrated a weight-adjusted dose of ST085384 or ST085405 in Cremaphor EL intraperitoneally to six-week old female C57BL/6J mice (*n =* 14*/*group, weight range 15–20 g). Inhibitor doses included 0 (Cremaphor EL, vehicle control), 10 and 100 mg/kg. All groups of mice were continuously monitored for signs of morbidity for a period of nine days. Collectively, these results shown in Figure 6 indicates that at a 100 mg/kg dose, ST085384 and ST085405 are non-toxic to mice, and are better tolerated than the general and potent kinase inhibitor, staurosporine (LD_50_ = 6.5 mg/kg [18]). These studies provide the foundation for future work to determine if kinase inhibitors that enhance MRSA susceptibility to β-lactams *in vivo*.

## 3. Discussion

*S. aureus* is one of five of the most common causes of nosocomial infections with an estimated 500,000 patients contracting a *S. aureus* infection each year [19]. As only 2% of all *S. aureus* isolates are sensitive to penicillin, this imposes significant constraints on treatment of these infections. A better understanding of mechanisms that increase antibiotic resistance in *S. aureus* is critical for development of new strategies.

In this study, we screened a kinase inhibitor library comprising low molecular weight sulfonamides and identified four inhibitors (ST085384, ST085404, ST085405, ST08539969) that increased the sensitivity of MRSA to β-lactams *in vitro*. Additional compounds that were structurally similar to the inhibitors exhibited reduced or minimal activity in our screen. This may be due to differences in cell permeability, or due to poor affinity of these compounds for MRSA Stk1. Future work, including purification and crystallization of Stk1 in complex with the effective inhibitors, is necessary to understand the exact mode of interaction.

Recently, Pensinger *et al.* [20] indicated that the general kinase inhibitor staurosporine did not increase sensitivity of MRSA to ceftrioxone leading to the notion that pharmacological inhibition of Stk1 may not be useful for MRSA. In contrast, we observed that staurosporine and the kinase inhibitors (ST085384, ST085404, ST085405, ST08539969) increased the sensitivity of MRSA to β-lactams such as Nafcillin. Additionally, in contrast to Pensinger *et al.* [20], we observed that staurosporine decreases autophosphorylation of *S. aureus* Stk1 *in vitro* (Figure 4). We speculate that the differing results on the role of staurosporine in Stk1 autophosphorylation may, in part, be due to one or all of the following: we used a full length his-tagged Stk1 whereas Pensinger *et al.* [20] used a GST tagged Stk1 kinase domain protein; other small differences were in reaction conditions and the composition of kinase buffer (see experimental methods and Pensinger *et al.* [20] for details). Nevertheless, our findings and previous studies by Tamber *et al.* [7] corroborate the notion that the kinase domain of the eukaryotic-like serine/threonine kinase regulates antibiotic resistance in MRSA. Consistent with these observations, inhibition of a kinase homologue also led to increased β-lactam susceptibility in *Listeria monocytogenes* [20], suggesting that there may be a common underlying mechanism. In contrast, varying results were obtained on the effect of kinase inhibitors that inhibit the essential serine/threonine PASTA kinases and growth of *Mycobacterium tuberculosis* [21,22]. However, a recent study indicated that the extracellular PASTA domain of serine/threonine kinases is essential for peptidoglycan remodeling and growth of *M. tuberculosis* [23]*.* This requirement of the PASTA domain of serine/threonine kinases in *M. tuberculosis*, rather than the kinase domain (as seen in MRSA) may, in part, contribute to the discrepancy in the effect of kinase inhibitors in various microorganisms.

We also observed that administration of two of the identified inhibitors (ST085384, ST085405) at 100 mg/kg dose did not induce any obvious morbidity or mortality in mice. However, further studies demonstrating the efficacy of the kinase inhibitors *in vivo* and establishing the lack of adverse symptoms at these concentrations are necessary to develop efficacious and safe inhibitors. In summary, the identification of kinase inhibitors that increase sensitivity of MRSA to β-lactams *in vitro* provides an avenue for exploration of alternative therapeutic strategies that can potentially decrease the severity of MRSA infections.

## 4. Experimental Section

### 4.1. Strains and Chemicals

Wild-type (WT) MRSA strains used in this study are clinical isolates MW2 (USA400) and LAC (USA300) [9]. Stk1 mutants in LAC and MW2 were constructed as described [8].

All chemicals were purchased from Sigma Aldrich. The kinase inhibitor library and the kinase inhibitors ST085384 and ST085405 were purchased from TimTec (Newark, NJ, USA, http://www.timtec.net/).

### 4.2. STK Inhibitor Library Screen

The kinase inhibitor library consisting of 1 mg each of 280 drug-like, low molecular weight sulfonamide compounds that can inhibit protein kinases were obtained from TimTec in a 96-well format (http://www.timtec.net/kinase-modulators-actitarg-k-library.html). Kinase inhibitors were re-suspended in DMSO (1 mg/mL). Overnight cultures of MRSA were subcultured 1:20 in tryptic soy broth (TSB), Nafcillin (NAF) was added to a final concentration of 4 μg/mL and dispensed into 96-well plates (100 µL/well). The kinase inhibitor was added to a final concentration of 40µg/mL, and the plate was incubated at 37 °C overnight with shaking. Bacterial growth was measured by recording the change in optical density at 600 nm (∆_OD600nm_) from before (T_0_) and after overnight incubation (T_O/N_). Percent inhibition was calculated using the formula ∆_OD600nm_ of “no inhibitor” control (max growth) −∆_OD600nm_ of sample (inhibitor + NAF)/∆_OD600nm_ of “no inhibitor” (max growth) × 100. As a control, each plate contained un-inoculated wells where no growth was observed. Additional controls also included wells that contained TSB + NAF (4 μg/mL) inoculated with the MRSAΔ*stk1* strain. Compounds with activity above two standard deviations of the sample set were further investigated. To calculate the MIC of these putative inhibitors in the presence and absence of Nafcillin, *S. aureus* cultures were prepared as described above and exposed to varying concentrations of each putative inhibitor.

### 4.3. Time-to-Kill Assay

The MRSA strain LAC was grown overnight from single colony. The next morning, approximately 10^4^ CFU/mL of LAC was added to 500 µL of either TSB, TSB containing NAF (4 µg/mL), TSB containing kinase inhibitor ST085384 (40 µg/mL), or TSB containing both NAF (4 µg/mL) and ST085384 (40 µg/mL). As a control, approximately 10^4^ CFU/mL of LACΔ*stk1* (which is sensitive to NAF) was added to either 500 µL of TSB or 500 µL of TSB containing NAF (4 µg/mL). All cultures were grown in a 37 °C shaker at 220 rpm. Aliquots of the media were removed immediately after subculture (T_0_) and at 2, 4, 8, and 24 h post-inoculation and were serially diluted and plated on TSA. Plates were incubated for 24–48 h at 37 °C and viable CFU were counted. The experiment was repeated three times. The above experiment was repeated with LAC∆*stk1*-see Figure legend for details.

### 4.4. Protein Purification and in Vitro Phosphorylation Assays

The *S. aureus stk1* gene was cloned into the expression vector pET32ck [13] to generate a C-terminal His-tagged fusion. To this end, Stk1 was PCR amplified using High Fidelity PCR (Primestar, Clonetech, Mountain View, CA, USA), the primer pairs 6His-SaSTKBamH1F and 6His-SaSTKXhoIR, and *S. aureus* RN4220 genomic DNA as the template. Of note, we confirmed that the coding sequence of the *stk1* gene in *S. aureus* RN4220 [14] is 100% identical to that found in MRSA USA300 [15]. The PCR product was digested with the enzymes for which restriction sites were engineered in the primers and were cloned in frame into the multiple cloning site (MCS) of pET32CK to obtain C-terminal His_6_ fusion protein. The recombinant plasmid was sequenced and expression was induced in *E. coli* BL21DE3 with 1mM IPTG at 16 °C overnight, shaking until cultures reached OD_600_ = 0.8. Cells were harvested by centrifugation, and His-tagged fusion protein was purified from cell free extracts using nickel affinity chromatography as described by the manufacturer (Qiagen, Valencia, CA, USA; (https://www.qiagen.com/us/resources/resourcedetail?id=0de4b003-4521-4fdc-923d-d304bdaddf5b&lang=en)). An aliquot of the purified Stk1 protein was analyzed using 10% SDS-PAGE followed by staining with Coomassie Brilliant Blue (Sigma, St. Louis, MO, USA). The purified protein was quantified using Bradford’s Reagent (Sigma). Subsequently Stk1 autophosphorylation was performed as described [24]. Briefly, ~1 µg of purified Stk1 protein was incubated in kinase buffer (50 mM TrisHCl pH 7.5, 1 mM MnCl_2,_ 1 µM ATP) containing 10 µCi [γ ^32^P] ATP (PerkinElmer) and either DMSO (control) or Staurosporine (2 µM) or ST085384 (2 µM) for 15 min. Subsequently, all reactions were heated to 100 °C for 5 min. The samples were then analyzed on 12% SDS-PAGE and exposed to autoradiography.

#### Primer Sequence

6His-SaSTKBamH1F: 5′ TAGAGGATCCATGATAGGTAAAATAATAAATGAAC 3′
6His-SaSTKXhoIR: 5′ TGAACTCGAGTACATCATCATAGCTGACTTCTT 3′

### 4.5. Animal Studies

Ethical Approval: All animal experiments were approved by the Institutional Animal Care and Use Committee (protocol 13311) and performed using guidelines in the *Guide for the Care and Use of Laboratory Animals* (8th Edition) and ARRIVE. To test if ST085384 and ST085405 are tolerated *in vivo*, mice were given an intraperitoneal dose of inhibitor (dissolved in Cremophor:Ethanol:PBS at 1:1:4) at 0, 10, or 100 mg/kg. Mice were monitored for survival for nine days.
